# ngsJulia: population genetic analysis of next-generation DNA sequencing data with Julia language

**DOI:** 10.12688/f1000research.104368.1

**Published:** 2022-01-31

**Authors:** Alex Mas-Sandoval, Chenyu Jin, Marco Fracassetti, Matteo Fumagalli

**Affiliations:** 1 Department of Life Sciences, Imperial College London, London, UK; 2 Institute of population genetics, University of Veterinary Medicine Vienna, Vienna, Austria; 3 Department of Ecology, Environment and Plant Sciences, Stockholm University, Stockholm, Sweden; 4 School of Biological and Behavioural Science, Queen Mary, University of London, London, UK

**Keywords:** high-throughput sequencing data, population genetics, genotype likelihoods, Julia language, pooled sequencing, polyploidy, aneuploidy

## Abstract

A sound analysis of DNA sequencing data is important to extract meaningful information and infer quantities of interest. Sequencing and mapping errors coupled with low and variable coverage hamper the identification of genotypes and variants and the estimation of population genetic parameters. Methods and implementations to estimate population genetic parameters from sequencing data available nowadays either are suitable for the analysis of genomes from model

organisms only, require moderate sequencing coverage, or are not easily adaptable to specific applications. To address these issues, we introduce ngsJulia, a collection of templates and functions in Julia language to process short-read

sequencing data for population genetic analysis. We further describe two implementations, ngsPool and ngsPloidy, for the analysis of pooled sequencing data and polyploid genomes, respectively. Through simulations, we illustrate the performance of estimating various population genetic parameters using these implementations, using both established and novel statistical methods. These results inform on optimal experimental design and demonstrate the applicabil-

ity of methods in ngsJulia to estimate parameters of interest even from low coverage sequencing data. ngsJulia provide users with a flexible and efficient framework for ad hoc analysis of sequencing data.ngsJulia is available from: https://github.com/mfumagalli/ngsJulia

## Introduction

Population genetics, i.e. the study of genetic variation within and between groups, plays a central role in evolutionary inferences. The quantification of genetic diversity serves the basis for the inference of neutral ^1^ and adaptive ^2^ events that characterised the history of different populations. Additionally, the comparison of allele frequencies between groups (i.e. cases and controls) is an important aspect in biomedical and clinical sciences. ^3^

In the last 20 years, next-generation sequencing (NGS) technologies allowed researchers to generate unprecedented amount of genomic data for a wide range of organisms. ^4^ This revolution transformed population genetics (therefore also labelled as population genomics) to a data-driven discipline. Data produced by short-read sequencing machines (still the most accessible platform worldwide) consists of a collection of relatively short (approx. 100 base pairs) fragments of DNA which are then mapped or *de novo* assembled to form a contiguous sequence. ^4^ At each genomic position, all observed sequenced reads are used to infer the per-sample genotype (an operation called ‘genotype calling’) and the inter-samples variability, i.e. whether a particular site is polymorphic (an operation called ‘single-nucleotide polymorphism (SNP) calling’). ^5^

To this end, several software packages have been implemented to perform genotype and SNP calling from NGS data, the most popular ones being samtools/bcftools, ^6^ GATK, ^7^ and freeBayes. ^8^ When, on average, few reads map at each genomic position (a scenario referred to as ‘low-coverage’ or ‘low-depth’), genotypes and SNPs cannot be assigned with confidence due to the high data uncertainty. ^9^^,^^10^ Under these circumstances, statistical methods that integrate data uncertainty into genotype likelihoods and propagate it to downstream analyses have been proposed. ^5^ Software packages like ANGSD ^11^ and ngsTools, ^12^ among others reviewed by Lou *et al,* ^13^ implement a statistical framework to estimate population genetic metrics from low-coverage sequencing data. Similarly, an affordable generation of sequencing data from large sample sizes can be obtained via pooled sequencing experiments, where assignment of individual samples is typically not retained. ^14^ Several new and popular software for the analysis of pooled sequencing have been proposed in recent years. ^15^^,^^16^

Despite these advances, most of these implementations are either tuned and suitable for model organisms only (e.g., with haploid or diploid genomes) or not easily adaptable to novel applications. Therefore, an accessible computational framework for building and testing *ad hoc* population genetic analyses from NGS data is in dire need. Among programming languages, Julia ^17^ has emerged as both a powerful and easy-to-use dynamically typed language that is widely used in many fields of data sciences, including genomics. ^18^ While several Julia packages are currently available for both population genetic and bioinformatic analyses (e.g., BioJulia), to our knowledge, a suitable framework for custom population genetic analysis from NGS data is not available yet.

Here we present ngsJulia, a set of templates and functions in Julia language to process NGS data and create custom analyses in population genetics. To illustrate its applicability, we further introduce two implementations, ngsPool and ngsPloidy, for the analysis of pooled sequencing data and polyploid genomes, respectively. By extensive simulations, we show the performance of several methods implemented in these programs under various experimental conditions. We also introduce novel statistical methods to estimate population genetic parameters from NGS data and demonstrate their applicability and suggest optimal experimental design. We finally discuss further directions and purposes for ngsJulia and bioinformatics for NGS data analysis.

## Methods

### Implementation

ngsJulia was built in Julia language (Julia Programming Language, RRID:SCR_021666) and requires the packages ‘GZip’ and ‘ArgParse’. Auxiliary scripts to process output files were built in R ( R Project for Statistical Computing, RRID:SCR_001905) version 3.6.3 and require the package ‘getopt’. ngsJulia receives gzipped mpileup input files which can be generated using samtools. ^19^ Output files are in text file format and can be easily parsed for producing summary plots and for further analyses. Scripts for some downstream analyses are provided in ngsJulia.

### Operation

ngsJulia is compatible with all major operating systems and is maintained at https://github.com/mfumagalli/ngsJulia Documentation and tutorials are available via this GitHub repository and archived at Zenodo ^20^ at the time of writing. All analyses in the manuscript are performed in Julia language and R.

ngsJulia implements functions to read and parse gzipped mpileup files and to output gzipped text files on various calculations (e.g., genotype and allele frequency likelihoods) and estimations (e.g., allele frequencies), as requested by the user. ngsJulia also allows for several data filtering options, including on global and per-sample depth, proportion or count of minor allele, and base quality. Finally, several options for SNP and biallelic and triallelic polymorphisms calling are available.

### Nucleotide, genotype and allele frequency likelihoods

ngsJulia provides utilities to calculate nucleotide and genotype likelihoods, i.e. the probability of observed sequencing data given a specific nucleotide or genotype, ^21^ for an arbitrary ploidy level, as in Soraggi *et al.* ^22^ We now describe how such quantities are calculated in ngsJulia.

Following the notation in Soraggi *et al,* ^22^ for one sample and one site, we let O be the observed NGS data, Y the ploidy, and G the genotype. Therefore, G has values in $\left\{0,1,\ldots ,Y\right\}$ , i.e. the number of derived (or alternate) alleles.

In the simplest form, genotype likelihoods can be calculated by considering individual base qualities as probabilities of observing an incorrect nucleotide. ^21^ We adopt the calculation of genotype likelihoods for an arbitrary ploidy level $P\left(O|G,Y\right)$ as proposed in Soraggi *et al.* ^22^ From the genotype likelihoods with $P\left(O|G,Y=1\right)$ , the two most likely alleles are identified by sorting $P\left(O|G,Y=1\right)$ values after pooling all sequencing reads together across all samples. This operation will restrict the range of possible genotypes to biallelic variation only. Note that this calculation is still valid for monomorphic sites, although the actual assignment of the minor allele is meaningless.

We now describe how to estimate $F_{a,n}$ , the frequency of allele $a\in \left\{A,C,G,T\right\}$ at site n . Similarly to Kim *et al,* ^23^ the log-likelihood function for $F_{a,n}$ is given by:

$$\log\left(P\left(O_{n}|F_{a,n}\right)\right)=\sum_{i=1}^{C_{n}}P\left(O_{n}|c_{i,n}=a,Y=1\right)F_{n}$$ (1)

where $c_{i,n}$ is the i−th read at site n , and $C_{n}$ is the total depth across all samples. Function 1 is maximised to obtain a maximum likelihood estimate (MLE) of the sample allele frequency, ${\hat{F}}_{n}$ , either with a grid- or golden-section- search algorithm.

### SNP calling

To perform SNP calling, we implement a likelihood-ratio test (LRT) with one degree of freedom with null hypothesis $H_{0}:F_{n}=0$ and alternate hypothesis $H_{1}:F_{n}={\hat{F}}_{n}$ , as described by Kim *et al.* ^23^ Additionally, we develop a test for a site being biallelic or triallelic. The former can be interpreted as a further evidence of polymorphism, while the latter as a condition not to be met for the site being included in further estimations, as our models assume at most two alleles. The log-likelihood of site n being biallelic is equal to $P\left(O_{n}|G_{n}=\left\{i,j\right\},Y=1\right)$ while the log-likelihood being triallelic is equal to $P\left(O_{n}|G_{n}=\left\{i,j,z\right\},Y=1\right)$ , with i , j , and z being the most, second most, and third most likely allele with Y=1 (i.e. haploid genotype G ), respectively. An LRT with one degree of freedom can be conducted to assess whether $P\left(O_{n}|G_{n}=i,Y=1\right)$ is significantly greater than $P\left(O_{n}|G_{n}=\left\{i,j\right\},Y=1\right)$ , or the latter is significantly greater than $P\left(O_{n}|G_{n}=\left\{i,j,z\right\},Y=1\right)$ .

### Allele frequency, site frequency spectrum and association test from pooled sequencing data

Several estimation of population parameters from pooled sequencing data are implemented in ngsPool, a separate program which uses functions in ngsJulia. We now describe the statistical framework for the analysis of pooled sequencing data.

In case of data with unknown sample size, the MLE of the population allele frequency $F_{n}$ is calculated as in Equation 1 with $F\in \left(0,1\right)$ . With known sample size, the same equation is used to calculate sample allele frequency likelihoods $P\left(O_{n}|F_{n}=f\right)$ _,_^24^ for instance with $f\in \left\{0,1,\ldots ,M\times Y\right\}$ for M samples of equal ploidy Y . From these likelihoods, we can calculate both the MLE and the expected value with uniform prior probability as estimators of $F_{n}$ .

A simple estimator of the site frequency spectrum (SFS) from pooled sequencing data is obtained by counting point-estimates of $F_{n}$ across all sites. We propose a novel estimator of the SFS implemented in ngsPool. Under the standard coalescent model with infinite sites mutations, we let the probability of derived allele frequency F in a sample of N genomes $P\left(F=f\right)$ to be proportional to $1/f^{K}$ with $f\in \left\{1,\ldots ,N-1\right\}$ _._^25^ The parameter K determines whether the population is deviating from a model of constant effective population size. For instance, K=1 is equal to the expected distribution of $P\left(F\right)$ under constant population size, while K>1 models a population shrinking and K<1 population growth.

We optimise the value of K to minimise the Kullback-Liebrel divergence between the expected distribution of $P\left(F|K\right)$ and the observed SFS. The latter can be obtained by either counting ${\hat{F}}_{n}$ across all sites or by integrating over the sample allele frequency probabilities $P\left(F_{n}=f|O_{n}\right)\propto P\left(O_{n}|F_{n}=f\right)$ ( *i.e.* with a uniform prior distribution). A threshold can be set to ignore allele frequencies with low probability to improve computing efficiency and reduce noise. Within this framework, folding spectra can be generated in case of unknown allelic polarisation.

Finally, we introduce a strategy to perform association tests from pooled sequencing data. Similarly to Kim *et al,* ^23^ we propose an LRT with one degree of freedom for null hypothesis $H_{0}:f_{\text{cases}}=f_{\text{controls}}$ and alternate hypothesis $H_{1}:f_{\text{cases}}\neq f_{\text{controls}}$ . The likelihood of each hypothesis is calculated from $P\left(O_{n}|F_{n}=f\right)$ and, therefore, this strategy avoids the assignment of counts or per-site allele frequencies. A statistically significant LRT with one degree of freedom suggests a difference in allele frequencies between cases and controls, and possible association between the tested phenotype and alleles.

### Ploidy levels and test for aneuploidy

We now describe the statistical framework implemented in the program ngsPloidy to estimate ploidy levels and test for aneuploidy. When multiple samples are available, two scenarios can be envisaged: (i) all samples have the same ploidy, (ii) each sample can have a different ploidy (aneuploidy).

The log-likelihood function for a vector of ploidy levels ${\vec{Y}}_{M}=\left\{Y_{1}=y_{1},Y_{2}=y_{2},\ldots ,Y_{M}=y_{M}\right\}$ for M samples and N sites is defined as:

$$\log(P\left(O|Y={\vec{Y}}_{M}\right)=\sum_{m=1}^{M}\sum_{n=1}^{N}\log\left(\sum_{i\in \left\{0,1,\ldots ,Y_{m}\right\}}P\left(O_{n}|G_{m,n}=i,Y_{m}=y_{m}\right)P\left(G_{m,n}=i|Y_{m}=y_{m},F_{n}={\hat{F}}_{n}\right)\right)$$ (2)

With ${\hat{F}}_{n}$ being the MLE of allele frequency at site n . $P\left(O_{n}|G_{m,n}=i,Y_{m}=y_{m}\right)$ is the genotype likelihood while $P\left(G_{m,n}=i|Y_{m}=y_{m},F_{n}={\hat{F}}_{n}\right)$ is the genotype probability given the ploidy and allele frequency at site n .

Once ${\hat{F}}_{n}$ is estimated and the inbreeding is known (or under the assumption of Hardy-Weinberg Equilibrium (HWE)), then genotype probabilities are fully defined. Equation 2 is optimised by maximising the marginal likelihood of each sample separately, assuming independence among samples and sites.

With limited sample size, ${\hat{F}}_{n}$ is not a good estimator of the population allele frequency and therefore genotype probabilities may not be well defined. In the simplest scenario, genotype probabilities can be set as uniformly distributed, with all genotypes being equally probable. However, the assignment of alleles in into ancestral (e.g., wild-type) and derived (e.g., mutant) states is particularly useful to inform on genotype probabilities. Recalling Equation 2, we can substitute $P\left(G_{m,n}=i|Y_{m}=y_{m},F_{n}={\hat{F}}_{n}\right)$ with $P\left(G_{m,n}=i|Y_{m}=y_{m},F_{n}=E\left[F|K\right]\right))$ , where $E\left[F|K\right]$ is the expected allele frequency of $P\left(F=f|K\right)\propto 1/f^{K}$ , as introduced previously. Note that $E\left[F|K\right]$ does not depend on the sequencing data for each specific site n .

Note that, in practice, Equation 2 is a composite likelihood function, as samples and sites are not independent observations due to shared population history and linkage disequilibrium, respectively. A solution to circumvent this issue is to perform a bootstrapping procedure, by sampling with replacement segments of the chromosome and estimate ploidy for each bootstrapped chromosome. The distribution of inferred ploidy levels from bootstrapped chromosomes provides a quantitative measurement of confidence in determining the chromosomal ploidy. Moreover, it is not possible to calculate the likelihood of ploidy equal to one after SNP calling, as only putative heterozygous genotypes will be retained. Nevertheless, the identification of haploid genomes from sequencing data is typically trivial, as the observation of polymorphisms should easily rule out the case of Y=1 .

### Unknown or uncertain ancestral allelic state

So far, we assumed to know which allele can be assigned to an ancestral state, and which one to a derived state. However, in some cases, such assignment is either not possible or associated with a certain level of uncertainty due to, for instance, ancestral polymorphisms or outgroup sequence genome from a closely related species not being available. Under these circumstances, we extend our formulation by adding a parameter underlying the probability that the assigned ancestral state is incorrectly identified.

Let us define R as the ancestral state and a as any possible allele in $\left\{A,C,G,T\right\}$ . In practice, a can take only two possible values as we select only the two most likely alleles. We label this set of the two most common alleles as A and we assume that the true ancestral state is included in such set. The log-likelihood function of ploidy for a single sample m under unknown ancestral state is:

$$\log(P\left(O|Y_{M}=y_{m}\right)=\sum_{n=1}^{N}\log\left(\sum_{a\in A}\sum_{i\in \left\{0,1,\ldots ,Y_{m}\right\}}P\left(O_{n}|G_{m,n}=i,Y_{m}=y_{m}\right)P\left(G_{m,n}=i|Y_{m}=y_{m},R=a,F_{n}={\hat{F}}_{n}\right)P\left(R=a\right)\right)$$ (3)

Where $P\left(R=a\right)$ indicates the probability that allele a is the ancestral state, and it is invariant across sites. If $P\left(R=a\right)=0.5$ , then the equation refers to the scenario of folded allele frequencies, where each allele is equally probable to be the ancestral state.

Finally, note that in a sufficiently large sample size, the major allele is more probable to represent the ancestral state. This probability depends on the shape of the site frequency spectrum, and it is equal to the cumulative distribution of $P\left(F|K\right)$ evaluated at F=N/2 . We can extend Equation 3 to reflect this parameter uncertainty with a being the major allele in $P\left(R=a\right)$ .

### Test for aneuploidy

We introduce a novel test for aneuploidy. If all samples have the same ploidy y∈Y , then $Y_{i}=Y_{j}=y$ is true for all $\left(i,j\right)\in 1,2,\ldots ,M$ . We propose an LRT for aneuploidy with null hypothesis $H_{0}:\sup\vec{Y}=\left\{y_{1}=y,y_{2}=y,\ldots ,y_{M}=y\right\}$ and alternate hypothesis $H_{1}:\vec{Y}={\vec{Y}}_{\mathrm{MLE}}$ A large value of LRT is suggestive of aneuploidy. Statistical significance can be assessed with the LRT and $\left(M-1\right)$ degrees of freedom.

### Data simulation

To benchmark the performance of methods implemented in ngsJulia, we simulated NGS data following a strategy previously proposed by Fumagalli *et al* ^26^ available as a stand-alone R script. Briefly, individual genotypes are drawn according to probabilities depending on input parameters. The number of mapped reads at each position is modelled with a Poisson distribution and sequenced bases are sampled with replacement with a probability given by the quality score. As an illustration, the following code
Rscript simulMpileup. R --out test.txt --copy 2x10 --sites 1000 \\--depth 20 --qual 20 ---pool|gzip > test.mpileup.gz


will simulate 10 diploid genomes (--copy 2x10), 1000 base pairs each ( --sites 1000) with an average sequencing depth of 20 and base quality of 20 in *Phred* score ( --depth 20 --qual 20) from pooled sequencing ( --pool) with results stored in test.mpileup.gz file.

For the analysis of pooled sequencing data, we simulated 100,000 independent sites at sample sizes 20 , 50 , and 100 from a diploid population with constant effective population size of 10,000 . We imposed the average per-sample sequencing depth to be 0.5 , 1 , 2 , or 5 with an average base quality of 20 in *Phred* score. To assess the performance of ngsPool, we calculated bias and root mean squared error (RMSE) between the estimated value of the true value, either from the sample or the whole population. While both metrics measure the distance with the true value, the bias retains the direction of the error (i.e. over- or under-estimation). To quantify the accuracy of SNP calling, we calculated F1 scores (the harmonic mean of precision and recall rates).

To simulate data for association test, we assumed an equal number of cases and controls ( 150 ) and 200 SNPs, either causal or non causal. For non causal sites, cases and controls have the same population allele frequency of 0.10 . For causal sites, cases and controls have a population allele frequency of 0.09 and 0.04 , respectively. These conditions are derived assuming a high risk allele frequency of 0.1 , prevalence of 0.2 , genotypic relative risk for the heterozygote of 2 , genotypic relative risk for the homozygous state of 4 . The sample size simulated guarantees at least 80% power with a false positive rate of 0.10 .

To illustrate the usage of ngsPloidy, we simulated NGS data of genomes with different ploidy (one haploid, eight triploids, one tetraploid) at 1000 sites. NGS data was simulated assuming an average depth of 10 at haploid level. Code and simulated data sets analysed are available in ngsJulia GitHub repository.

## Results

ngsJulia implements data structures and functions for an easy calculation of nucleotide and genotype likelihoods (of arbitrary ploidy) which serve the basis of genotype and SNP calling and for the estimation of allele frequencies and other summary statistics. It is particularly suitable for low-coverage sequencing data and for cases when there is high data uncertainty. To demonstrate the use of ngsJulia, we provide two custom applications from its templates and functions.

### ngsPool: analysis of pooled sequencing data

We used ngsJulia to implement a separate program, called ngsPool, to perform population genetic analysis from pooled sequencing data. Specifically, ngsPool implements established and novel statistical methods to estimate allele frequencies and site frequency spectra (SFS) and perform association tests from pooled sequencing data.

ngsPool uses functions in ngsJulia to parse mpileup files as input. As an illustration, the following code


julia ngsPool.jl --fin test.mpileup.gz --fout test.out.gz --lrtSnp 6.64


will parse test.mpileup.gz file and write estimates of allele frequencies in test.out.gz file from unknwon sample size after performing SNP calling with an LRT value of 6.64 (--lrtSnp 6.64, equivalent to a *p*-value of 0.01 ).

Depending on the options selected by the user, output files contain, various results are printed on the screen, including

• inferred major allele,
• inferred minor allele,
• LRT statistic for SNP calling,
• LRT for bi- and tri-allelic calling,
• three estimators of the minor allele frequency at each site.

Additionally, ngsPool can output a file with per-site sample allele frequency likelihoods. The following code


julia ngsPool.jl --fin test.mpileup.gz --fout test.out.gz \\--nChroms 20 --fsaf test.saf.gz


will produce estimates of allele frequencies from known sample size (specified by --nChroms 20) and allele frequency likelihoods in test.saf.gz file.

These files can then be used to estimate the SFS and perform an association test using two scripts provided in ngsPool. For instance, the code


Rscript poolSFS. R test.saf.gz > sfs.txt


will estimate the SFS, while the code


Rscript poolAssoc. R test.cases.saf.gz test.controls.saf.gz > assoc.txt


will perform an association test assuming two sets of allele frequency likelihood files, one from cases ( test.cases.saf.gz) and one from controls ( test.controls.saf.gz).

### Estimation of allele frequencies

To illustrate the usage of ngsPool, we estimated allele frequencies based on simulated data at different experimental conditions. We sought to compare the performance among different estimators implemented in the program. If the sample size is not provided, ngsPool provides a simple MLE of the population allele frequency assuming haploidy. Alternatively, if the sample size is provided by the user, ngsPool calculates sample allele frequency likelihoods and returns both the MLE and the expected value of the allele frequency using a uniform prior probability.

**Figure 1.**
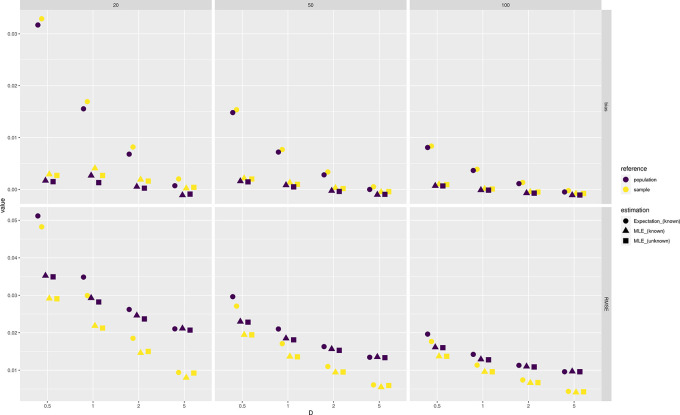
Estimation of minor allele frequencies from pooled sequencing data. Bias and root mean square error against the reference true value from either the population or sample are provided for each value of depth D (the average number of sequenced reads per base pair) and sample size (on column panels) tested. Three estimators of allele frequencies are considered: a population maximum likelihood estimate (MLE) from unknown sample size and the expected value and MLE from known sample size.

Results show that the error of estimating allele frequencies decreases with increasing depth and sample size, as expected ( Figure 1). Likewise, the error to estimate the population allele frequency are more pronounced for lower sample sizes. MLE values tend to be less biased than expected values and sample estimates appear to be unbiased even at depth 1 for moderate sample size ( Figure 1).

**Figure 2.**
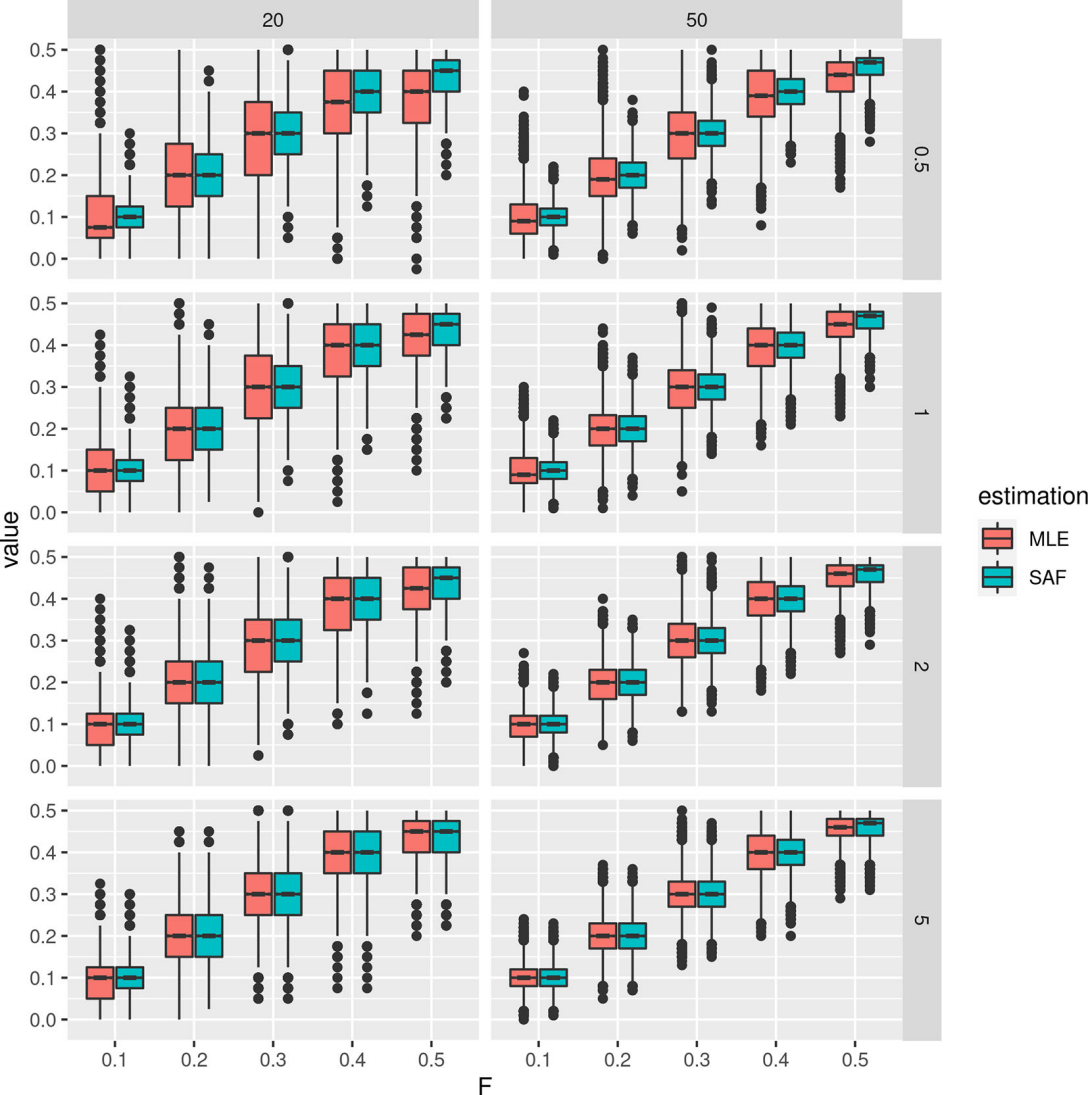
Distribution of estimated minor allele frequencies from pooled sequencing data. True sample allele frequencies and maximum likelihood estimates are shown at different fixed population allele frequencies F and sample sizes (on columns, 20 and 50) and depths (the average number of sequenced reads per base pair, on rows, 0.5, 1, 2, and 5).

Furthermore, we simulated NGS data at fixed population allele frequency and compared the distribution of true sample allele frequencies and estimated values using a MLE approach from known sample size. Figure 2 shows that most of the deviation from the true population allele frequencies occur at intermediate frequencies ( F equal to 0.5). This effect is more evident for low depth and low sample size ( Figure 2).

**Figure 3.**
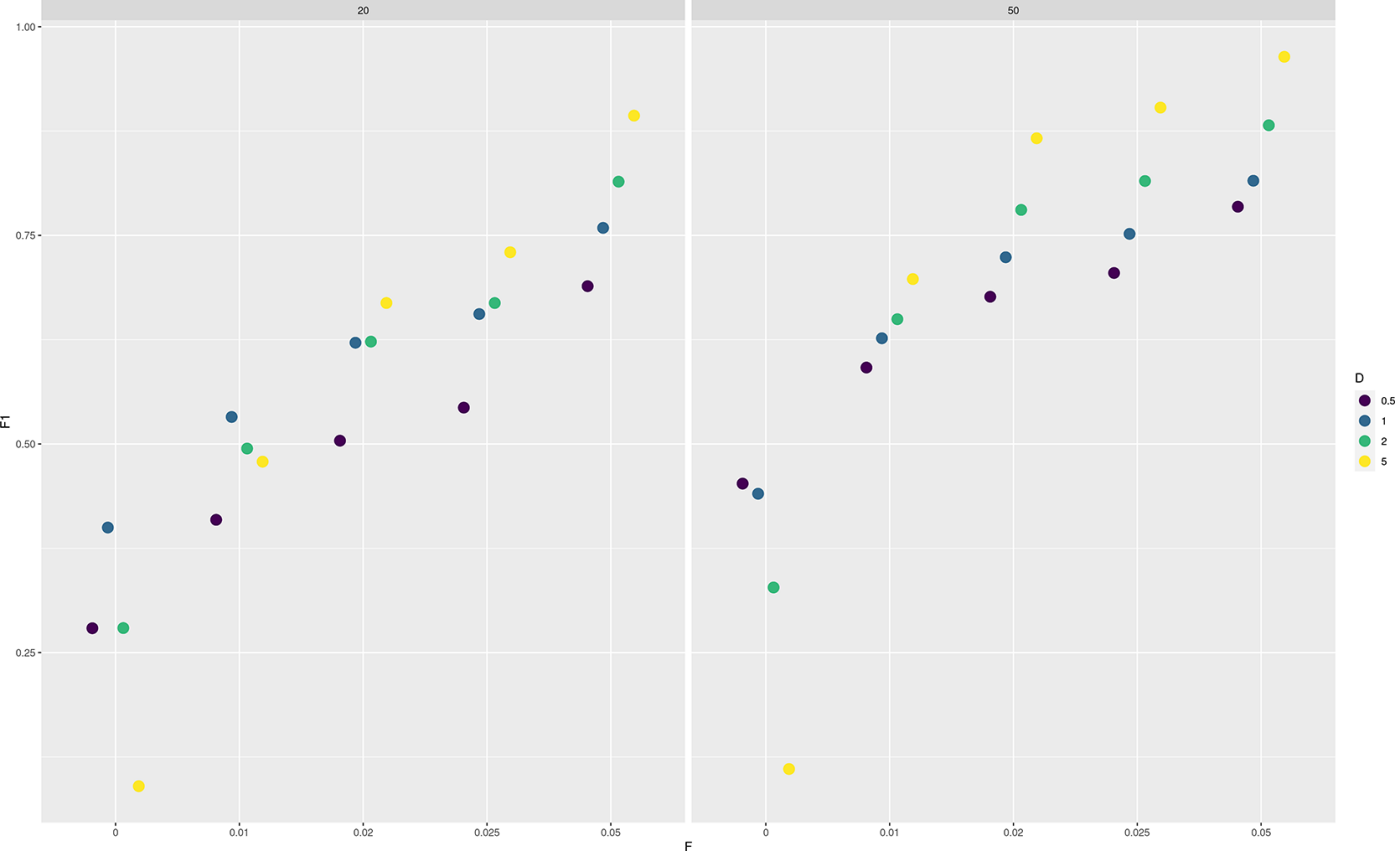
Accuracy of single nucleotide polymorphism (SNP) calling from pooled sequencing data. F1 scores (harmonic means of precision and recall rates) for predicting either a SNP (true population allele frequency F greater than 0) or not ( F=0 ) are reported are various values of F , sample sizes (on columns, 20 and 50) and depths D (the average number of sequenced reads per base pair).

We then assessed the effect of low-frequency variants on SNP calling. Specifically, we calculate F1 scores for SNP calling when the population allele frequency is 0 (not a SNP) or greater than 0 (a SNP). Figure 3 shows how the prediction accuracy increases with the population allele frequency ( F ), sample size, and depth ( D ). For instance, an F1 score greater than 0.75 is obtained with 20 samples only for F=0.05 and D>0.5 . On the other hand, the same F1 score is achieved with 50 samples even with F=0.025 and also at F=0.02 but only if D>1 .

SNP calling under-performs when there is no variation in the population ( F=0 ), with sequencing errors and sampling statistical uncertainty generating estimate of F greater than 0.

ngsPool implements several methods to estimate the SFS from sample allele frequencies. As described in the methods, a simpler estimator is based on assigning the most likely sample allele frequency at each site (labelled count). ngsPool implements novel estimators of SFS from pooled sequencing data as described in the method section. A script implements an algorithm to fit the theoretical SFS to the observed SFS. The latter can be calculated either by assigning per-site MLE of allele frequencies (labelled fit_count) or by integrating the uncertainty across all sample allele frequency likelihoods (labelled fit_afl).

**Figure 4.**
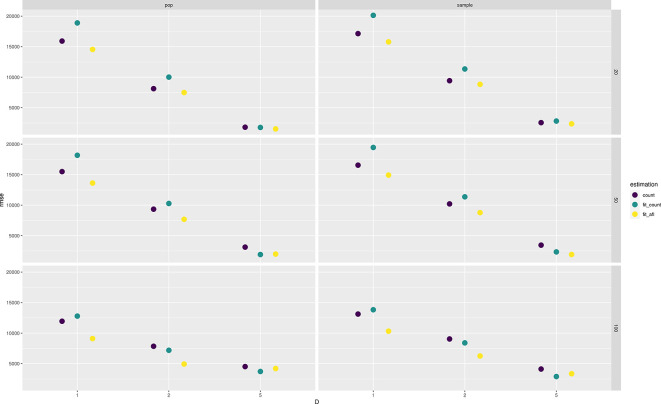
Estimation of site frequency spectrum (SFS) from pooled sequencing data. Root mean square error (RMSE) values between true (either population or sample) and estimated SFS are reported at various depths D and sample sizes (on the rows).

Figure 4 shows the error in estimating either the population or sample SFS at various settings with different methods. The error decreases with increasing depth and sample size. Estimating SFS by fitting the theoretical SFS without assignment of allele frequencies generally outperforms other tested strategies ( Figure 4).

**Figure 5.**
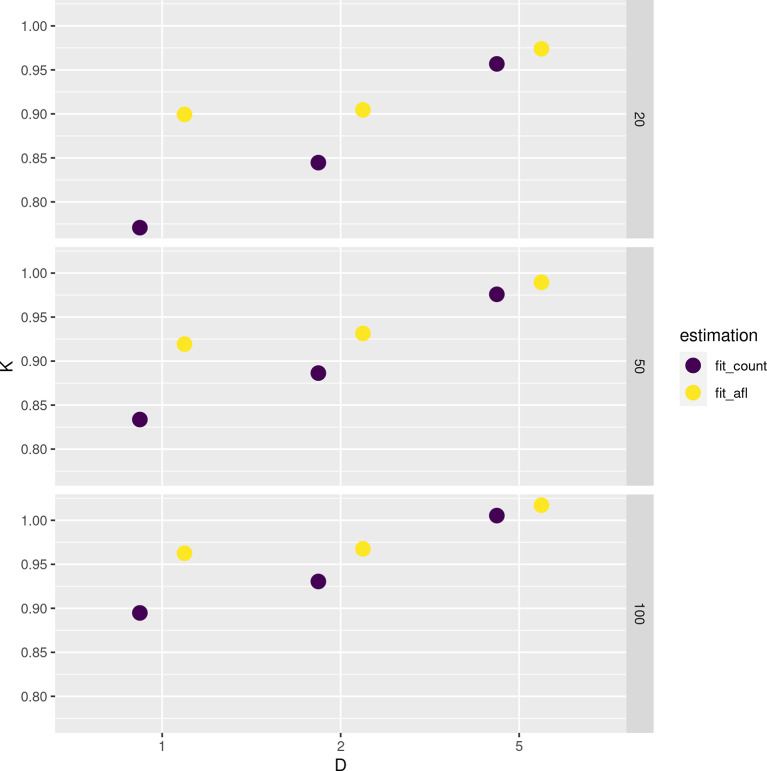
Estimation of parameter K (used to determine if the population is deviating from constant effective population size) of the site frequency spectrum at different depths D and sample sizes (on rows) from pooled sequencing data. Estimates based on fitting from counted allele frequencies ( fit_count) and from allele frequency likelihoods ( fit_afl) are reported. Note that the true simulated value of K is 1 .

Notably, the novel approach implemented in ngsPool to estimate the parameter K of the SFS distribution (see Methods) allow us to directly quantify the error in inferring demographic events. In fact, all simulations assumed constant population size, equivalent to K=1 . Figure 5 shows the estimated values of K by either assigning (counting) allele frequencies ( fit_count) or by using allele frequency likelihoods ( fit_afl). For low-to-moderate depth and sample size, estimates of K tend to suggest population expansion ( K<1 ), possibly due to an over-estimation of the abundance of low-frequency alleles. However, the error is reduced when integrating the data uncertainty with sample allele frequency likelihoods, as estimates of K values tend to be closer to the true simulated value of 1 ( Figure 5).

Estimation of minor allele frequencies from pooled sequencing data. Bias and RMSE against the reference true value from either the population or sample are provided for each value of depth D and sample size (on column panels) tested. Three estimators of allele frequencies are considered: a population MLE from unknown sample size and the expected value and MLE from known sample size.

ngsPool implements a script to perform association tests from pooled sequencing data. Specifically, the script calculates an LRT statistic, with null hypothesis being that allele frequencies of cases and controls (or any two groups) are the same, as used by Kim *et al.* ^23^ It uses sample allele frequency likelihoods and, therefore, it maintains data uncertainty and avoids the assignment of counts or per-site allele frequencies. An LRT statistics significantly greater than 0 indicates a difference in allele frequencies between cases and controls.

**Figure 6.**
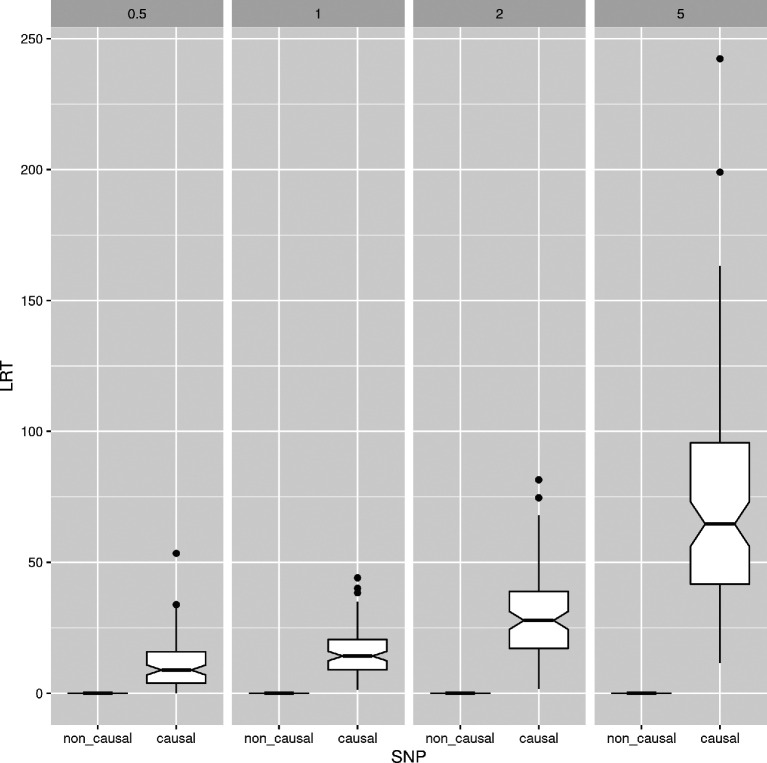
Performance of association test from pooled sequencing data. The distribution of likelihood-ratio test (LRT) statistics for casual and non causal single-nucleotide polymorphism (SNP) is reported at different depths (the average number of sequenced reads per base pair, on columns).

Figure 6 compares the distribution of LRT statistics between causal and non causal sites at different experimental scenarios. The distribution of LRT at causal SNPs is skewed towards higher values for increasing depth, indicating more support to find phenptype-SNP association. Nevertheless, a clear separation between the distributions of causal and non causal SNPs is observed at low depth ( Figure 6).

### ngsPloidy: analysis of sequencing data from polyploid genomes

We further utilised ngsJulia to implement an additional program, ngsPloidy, for the estimation of ploidy from unknown genotypes. The method implemented is similar to the one proposed by Soraggi *et al* ^22^ with some notable differences on the calculation of genotype probabilities (see Methods). Additionally, ngsPloidy includes a novel method to test for aneuploidy in the sample. As an illustration, the following code


julia ngsPloidy.jl --fin test.mpileup.gz --nSamples 20 > test.outRscript ploidyLRT. R test.out


will estimate ploidy levels for 20 genomes ( --nSamples 20) from test.mpileup.gz file and return LRT values.

Following Equation 2, the genotype probabilities for each tested ploidy are pre-calculated using a script provided in ngsPloidy. This script takes as input the value of parameter K (the shape of the expected SFS), the effective population size, and the probability that the major allele is the ancestral allele. The latter can be either set by the user (e.g., a value of 0.5 would be equivalent to unknown polarisation, as in a folded SFS) or be calculated from the expected population SFS itself. If genotype probabilities are not set, then a uniform distribution is assigned. Further options allow for estimation only on called SNPs and/or genotypes.

ngsPloidy uses functions in ngsJulia to parse mpileup files as input. At the end of the computation, various results are printed on the screen, including

• number of analysed sites that passed filtering for each sample,
• a matrix of ploidy log-likelihoods for each sample,
• the log-likelihood and MLE of the ploidy vector (i.e. the individually estimated ploidy for each sample),
• LRT scores for the test of aneuploidy against all tested ploidy levels.

Additionally, if requested by the user, ngsPloidy can generate output files with several statistics for each site (e.g., estimate allele frequency), and all per-site genotype likelihoods for each sample and tested ploidy.

To illustrate the usage of ngsPloidy, we deployed it to simulated data of an aneuploid sample consisting of one diploid, eight triploid and one tetraploid genomes. We compared the performance of ploidy and aneuploidy inference among different choices of genotype probabilities. The latter were derived either from the expected folded population site frequency, from the estimated ancestral population allele frequency, or from the calculated sample allele frequency.

In all tested cases, we inferred the correct vector of marginal ploidy levels. We therefore assessed the confidence in such inference by calculating the LRT statistics of ploidy and aneuploidy inferences. Both were calculated by comparing the most against the second most likely vector of ploidies or the most likely vector of equal ploidies, respectively.

Results show that using the per-site estimate sampled allele frequency yields higher LRT statistics (and therefore confidence) than using expected population allele frequencies ( Table 1). We reiterate that for all three cases we correctly identified the patterns of aneuploidy. However, we should caution that with lower sample sizes we do not expect inferences using estimated sample allele frequencies to perform well.

**Table 1.** Confidence values to assigned the correct ploidy vector and test for aneuploidy. Likelihood-ratio test (LRT) statistics using different methods of incorporating allele frequencies are reported.

Allele frequency	LRT – ploidy	LRT - aneuploidy
Folded	34.19	252.72
Ancestral	31.49	277.91
Sample	37.83	373.07

## Discussion

Analyses presented here provide further support for the use of genotype and allele frequency likelihoods in the analysis of NGS data. ^5^ Notably, we demonstrated how probabilistic estimates of population genetic parameters can be obtained in case of pooled sequencing data and short-read data from polyploid genomes. Additionally, we motivated the inference of SFS from allele frequency likelihoods as a direct way to infer demography from raw sequencing data.

ngsJulia offers new possibilities of software prototyping for custom analyses of NGS data for population genetic applications. Furthermore, it allows for efficient testing of experimental designs and, therefore, would be beneficial for initial planning of any sequencing experiments. Finally, ngsJulia is highly applicable in educational contexts, with accessible documentation and tutorials to educate users on the theory underpinning the implemented methods. We envisage that further improvements in ngsJulia will include the expansion of suitable input formats and data file types, and the compatibility with additional NGS data type, including from long-read sequencing experiments. ^27^

## Conclusions

In this study, we introduce ngsJulia, a series of templates and functions in Julia language to analyse NGS data for population genetic purposes. We present two implementations for the analysis of pooled sequencing data and polyploid genomes, with the inclusion of novel methods. ngsJulia is a suitable framework for prototyping new software and for custom population genetic analyses from NGS data.

## Data availability

### Underlying data

Simulated data and pipeline to reproduce all results presented here are available at https://doi.org/10.5281/zenodo.5886879. ^20^

Data are available under the terms of the Creative Commons Attribution 4.0 International Public License.

## Software availability

• Source code available from: https://github.com/mfumagalli/ngsJulia
• Archived source code at time of publication: https://doi.org/10.5281/zenodo.5886879 ^20^
• License: Creative Commons Attribution 4.0 International Public License
