# Supplementary material for: ngsJulia: population genetic analysis of next-generation DNA sequencing data with Julia language
Source: F1000Res. 2023 Jul 14;11:126. Originally published 2022 Jan 31. [Version 3] doi: 10.12688/f1000research.104368.3 (PMC10514575; doi:10.12688/f1000research.104368.3)
Supplement: Peer review history

# Reviewer report: Reviewer response for version 3

**DOI:** 10.5256/f1000research.150492.r202079

**Published:** 2023-10-11

**Authors:** Jens Léon, Michael Schneider

**Affiliations:** 1 University of Bonn, Bonn, Germany; 2 Forschungsinstitut fur Biologischen Landbau, Frick, Aargau, Switzerland

First of all, I have to write that we are working on a method to estimate the allele frequency in crops by pool sequencing. Therefore, we are very interested in this new methodology. In this review our research group have a look on the manuscript. We are researchers dealing with population and quantitative genetics of plants and other are dealing with bioinformatics.

 We recognized that the examples and sources for the testing are rather complex. The authors explain their probes to be haploid, usually diploid but also polyploid. They state that the samples of the organisms are mostly biallelic but sometimes also tri-allelic. We expect that in a population we usually have multiple alleles. The authors are also dealing with pooled samples. But for researches dealing with theses probes, it is rather confusing to understand the restrictions. Regarding a divers readership, I would like to recommend that the authors clearly define their samples first (haploid, diploid or polyploid and whether biallelic or multi-allelic loci are expected). When dealing with pooled samples the readership should know about the expectation of the samples (e.g. pool of sperms or pollen grains are different from a pool of cells from an individuum). Analysing populations with pooled NGS, it would be important to know how the software deals with a highly heterogenous material, as several individuals in a population. Although the authors tried to group their methods, it is still unclear which expectations the authors have to the samples.

 It would be helpful to provide a code example of creating the mpileup file.

 Why do you need to provide a new tool to call SNPs? The three tools in the introduction are all solid options

 and can give allele frequency calls, too. Adding the info on the expected allele frequency is helpful - but the effect of this additional information

 would be worth demonstrating. E.g., by showing the accuracy of recalling the correct allele frequency with and without this term. Besides,

 how would someone with a heterogeneous population set this value?

 It would be helpful to illustrate the actual distribution of reads across the genome. It is hard to imagine that with a uniform coverage of 0.5,

 which is not uncommon these days to receive from a WGS run, a good recall rate of the allele frequency can be achieved. It would, therefore, be helpful to understand the assumed input data better and provide the lambda value of the Poisson model for read simulations.

 There is no information provided about the recall rate of simulated SNPs. How many SNPs were detected on the different sequencing depth levels,

 and how many were falsely discovered? This is a major problem in the other named variant callers at very low sequencing depth levels, as discussed

 in this manuscript, and it would be of high value to compare the accuracy and sensitivity of the new ngsPool approach and the "old" once, like bcftools

 and GATK.

 Real Arabidopsis data comparing ngsPool to VarScan. As previously mentioned, why not compare also to GATK, FreeBayes, and bcftools? VarScan to my knowledge is not one of the top tools in terms of variant detection.

 To give this manuscript more relevance and make the decision to use it or not more accessible for the reader, I would encourage the authors to provide the information if this new package performs more precisely than common tools, if not, or under which circumstances it does. If the accuracy is not improved, what are the other benefits? Time-saving, user-friendly output.

 Figure caption of Figure 1: the order in the graphics is not the same as the one in the description. That can be changed easily and helps the reader.

Are the conclusions about the tool and its performance adequately supported by the findings presented in the article?

Yes

Is the rationale for developing the new software tool clearly explained?

Yes

Is the description of the software tool technically sound?

Partly

Are sufficient details of the code, methods and analysis (if applicable) provided to allow replication of the software development and its use by others?

Partly

Is sufficient information provided to allow interpretation of the expected output datasets and any results generated using the tool?

Partly

Reviewer Expertise:

Population and quantitative genetics of crops, Plant breeding, cereals

We confirm that we have read this submission and believe that we have an appropriate level of expertise to confirm that it is of an acceptable scientific standard, however we have significant reservations, as outlined above.

# Reviewer report: Reviewer response for version 3

**DOI:** 10.5256/f1000research.150492.r202073

**Published:** 2023-09-21

**Authors:** Nikolay Oskolkov

**Affiliations:** 1 Department of Biology, National Bioinformatics Infrastructure Sweden, Science for Life Laboratory, Lund University, Lund, Sweden

The authors suggest a population genomics tool written in Julia programming language. They demonstrate the performance of the tool on simulated and real pooled sequencing data. The manuscript is well written and technical details of the tool are well explained. In addition, documentation is provided together with the tool.

 I believe, it is potentially interesting to approach some bioinformatics analyses (such as population genomics) with Julia. However, I am still missing a clear motivation for re-writing e.g. genotype likelihoods and SFS computation in Julia. There are many popgen tools that are fast and accurate enough, e.g. ANGSD, so there should be a clear advantage of ngsJulia in some way compared to other tools. If the motivation is the outstanding speed or accuracy of ngsJulia, it should be clearly demonstrated. So my main concern is the lack of comparison of ngsJulia with other popgen tools.

 Next, I would not advertise ngsJulia as a “ *population genetic analysis of next-generation sequencing data*”, as from the text I got an impression that this was a quite pool-seq-specific tool. If ngsJulia is more general than a pool-seq data tool, it should be demonstrated and compared with other tools. Otherwise, I would suggest to change the title of the manuscript and make it less general and more specific. For example, accounting for polyploidy is a strength of ngsJulia, perhaps this should be emphasized more in the manuscript.

 I found it interesting and novel to determine the optimal K in the 1/f^K approximation of SFS profile with Kullback-Leibler optimization. However, as it seems to me from the Figure 5, this implementation in ngsJulia only works for D ~ 5 and large sample sizes. Perhaps other tools will not do a great job either, but as I mentioned above, I am missing the comparison with other tools.

 Minor comment:

 Page 7 I would encourage the authors to make the command line for ngsPool.jl more self-explanatory. More specifically, the “ *–lrtSnp 6.64*” flag is hard to grasp. Perhaps re-writing it in a way so that it becomes clear that 6.64 corresponds to p-value = 0.01, and replacing the flag “–lrtSnp 6.64” with “--pvalue 0.01” would be possible?

Are the conclusions about the tool and its performance adequately supported by the findings presented in the article?

Partly

Is the rationale for developing the new software tool clearly explained?

Partly

Is the description of the software tool technically sound?

Yes

Are sufficient details of the code, methods and analysis (if applicable) provided to allow replication of the software development and its use by others?

Yes

Is sufficient information provided to allow interpretation of the expected output datasets and any results generated using the tool?

Yes

Reviewer Expertise:

bioinformatics, genomics, medical genetics, ancient DNA

I confirm that I have read this submission and believe that I have an appropriate level of expertise to confirm that it is of an acceptable scientific standard, however I have significant reservations, as outlined above.

# Reviewer report: Reviewer response for version 3

**DOI:** 10.5256/f1000research.150492.r187509

**Published:** 2023-08-14

**Authors:** Stéphane De Mita

**Affiliations:** 1 Institut national de la recherche agronomique (INRAE), Paris, France

After considering the updated version and using the new documentation, I managed to run some tests successfully. However, I couldn't understand the return value of calcGenoLike without extrapolating from what it done in the triploid case in the tutorial.

Are the conclusions about the tool and its performance adequately supported by the findings presented in the article?

Yes

Is the rationale for developing the new software tool clearly explained?

Yes

Is the description of the software tool technically sound?

Yes

Are sufficient details of the code, methods and analysis (if applicable) provided to allow replication of the software development and its use by others?

Partly

Is sufficient information provided to allow interpretation of the expected output datasets and any results generated using the tool?

Partly

Reviewer Expertise:

Population genomics, phytopathology, programming (Python, C, C++).

I confirm that I have read this submission and believe that I have an appropriate level of expertise to confirm that it is of an acceptable scientific standard.

# Reviewer report: Reviewer response for version 2

**DOI:** 10.5256/f1000research.141471.r160519

**Published:** 2023-02-21

**Authors:** Stéphane De Mita

**Affiliations:** 1 Institut national de la recherche agronomique (INRAE), Paris, France

The article "ngsJulia: population genetic analysis of next-generation DNA sequencing data with Julia language" by Alex Mas-Sandoval and colleagues provides a library for the Julia addressing management of NGS data (provided in the PileUp format), SNP and genotype calling allowing for arbritary and dataset-varying ploidy, and maximum-likelihood parameter estimation.

 I've really discovered the Julia language while reviewing this article and I agree that it makes sense to use this language for developing tools for the analysis of NGS data. Providing a toolkit to perfom SNP and genotype calling under a probabilistic models makes much sense and can be useful for the community. The ngsPool application provided can be of widespread use while ngsPloidy can be a very precious tool for specific systems which lack proper software right now.

 Here is a list of random typos or detail-level suggestions:

- "polidy" in the "Revision" box.
- Methods: you should add Combinatorics as a requirement.
- I am not sure to understand the header "Operation".
- One or two sentences, presenting biological systems where the study of multiploidy (or in general MLE of ploidy) is relevant, would help.
- I am not sure giving an example of a R command line for simulating data is a good choice. For a Julia package I'd like to see some Julia code example, although it is not absolutely necessary.
- Discussion: rewrite: "is highly applicable in educational contexts"

I do not get this sentence at all: " *[ngsJulia] allows for efficient testing of experimental designs and, therefore, would be beneficial for initial planning of any sequencing experiments.*"

 My big issue with the paper, which is confirmed by a first look at the package, is that the content of the ngsJulia library isn't described, even succinctly. There should be an API and the article should point to it.

 In my view there is still need for more documentation. Tutorials are great and (speaking by experience) they are often essential, but at some point you need for some more rigourous reference documentation (API for the library, CLI for the applications). The Application Programmer Interface should be documented so that the reading planning to write a script using the library can know what functions are available, what input they expect, and if possible what errors can be expected. This remark also applies to the Command Line Interface of the two applications ngsPloidy and ngsPool. The help page produces an option summary, which is fine, but the meaning of the options isn't documented.

 Below I report the results of my testing of the three components of the software.

 *** ngsPloidy ***

 The tutorial is a good resource to start, but some points need clarification, and a more rigourous reference manual of the command-line interface would help a lot.

 I am not sure that it is a good idea to start by defining environment variables, which make the commands much less friendly. At least, do mention that you are defining environment variables so that unaware users can know where to look at if they need more information. Anyway the julia command should be available is it is installed in the environment which should be considered to be the default. By the way, "Julia language" is a poor choice of words.

 I am sorry, but I didn't get at all what "genotype probabilities" where and whether and why would I need to to create them. I have no idea what writePars is doing and -p and -s arguments aren't clearly documented.

 There is a "variabile" (instead of "variable") in the in simulMpileup.R manual. The simulation script needs its own documentation.

 Personally, I would think that either bam files containing aligned reads, or VCF, would be a better choice than PileUp, because users are more likely to already have those.

 Suggestions: you could print progress information while running, and displays error messages in stdout instead of stderr (especially useful since you advice users to redirect results which are printed in stdout.

 I tested ngsPloidy using data from my own read simulator. I thought it was a good idea to bring data from an independent source, and also I am more comfortable using it. It generated data from diploid and tetraploid organisms, using a coalescent model to draw allelic frequencies.

 Then I ran this type of commands:

 $ julia ../ngsJulia/ngsPloidy/ngsPloidy.jl --fin diplo.plp.gz --nSamples 20 > diplo.out

 Running times were (tens of minutes up to an hour for rather small simulated datasets). I suspect that I should have dropped non-varying sites from the PileUp (since I didn't incorporate errors, 90% of my sites are completely fixed).

 When I simulated only diploid or tetraploid individuals, they are completely inferred (although in some parameter settings I had tetraploid individuals estimated as a mixture of tetra- and pentaploid individuals). When I merge diploid and tetraploid individuals in a single PileUp, the software tended to estimate a mixture of diploid and triploid individuals. So I suspect that I am doing something wrong. Most likely I miss an important step of the procedure and the documentation might be to blame.

 *** ngsPool ***

 Among the command line arguments, by "LRT", do you mean "threshold"?

 I got an exception when the pileup file didn't contain reference bases. I understand that this should be an error but it could be displayed in a more accessible manner.

 Some test results are reported as "Inf" or "-Inf" which is also not necessarily clear.

 I was not totally clear when --lrt* arguments should be used.

 I got a problem to understand the difference between maf, saf_MLE and saf_E? It is also a problem with the article, where the logic is mentioned by not really explained.

 I ran a simulation with a 20,000 bp genome, 20 diploid individuals, 10% of sites with diallelic polymorphism, 10,000 reads of 50 bp (so an average depth of 25X).

 I ran:

 # julia ../ngsJulia/ngsPool/ngsPool.jl --fin data.plp.gz \

             --fout results.txt.gz  --lrtSnp 6.64 

 It took ~ 4:30 minutes and the results are fitting the simulation parameters. All sites called as SNPs are correct and only occasionnaly a few number (~1/1000) of "real" SNPs aren't called, and whose are always singletons. There is also a good agreement between the 'real' and estimated maf.

 Using the --nChroms option gives similar results and all estimated frequencies are correct.

 *** ngsJulia ***

 An API reference  was badly needed. I tried to start writing a Julia script analysing one of the datasets used for previous testing and didn't get very far. My lack of knowledge of Julia didn't help, for sure.

 The last paragraph of the readme "In general, ngsJulia provides templates..." should come first.

 Is it normal that we need to import Combinatorics even if we are not directly using it (i.e. I reckon that ngsJulia should import it itself).

 Almost instantly, I have the following line crashing:

   nucleoLikes = [calcGenoLike(myReads, [i], 1) for i=1:4]

 The error message is: ERROR: LoadError: BoundsError: attempt to access SubString{String}

   at index [2]

 I thought it was because I was trying to pass a site (represented by a Reads object, I assume), containing a single read base. But in the end it is not this (sites with more bases are not accepted either). Obviously I am making a very basic error but the lack of documentation doesn't help me.

Are the conclusions about the tool and its performance adequately supported by the findings presented in the article?

Yes

Is the rationale for developing the new software tool clearly explained?

Yes

Is the description of the software tool technically sound?

Yes

Are sufficient details of the code, methods and analysis (if applicable) provided to allow replication of the software development and its use by others?

Partly

Is sufficient information provided to allow interpretation of the expected output datasets and any results generated using the tool?

Partly

Reviewer Expertise:

Population genomics, phytopathology, programming (Python, C, C++).

I confirm that I have read this submission and believe that I have an appropriate level of expertise to confirm that it is of an acceptable scientific standard, however I have significant reservations, as outlined above.

# Reviewer report: Reviewer response for version 1

**DOI:** 10.5256/f1000research.114499.r128232

**Published:** 2022-04-13

**Authors:** Lindsay V. Clark

**Affiliations:** 1 Roy J. Carver Biotechnology Center, University of Illinois at Urbana-Champaign, Urbana, IL, USA

The manuscript by Mas-Sandoval et al. describes ngsJulia, a collection of functions and scripts in Julia and R for estimating genotype likelihoods from short read DNA sequencing data, as well as estimating allele frequencies in pooled samples, and estimating ploidy where it is unknown. It seems that the goal is also to enable biologists proficient in Julia to develop their own custom genotype calling and population genetic analysis scripts starting with the provided functions for estimating genotype likelihoods. As it is, the manuscript and software suffer from two major flaws: (1) the software and its outputs are not well documented for users or developers, and (2) testing is only performed on simulated data, not empirical data.

 The software documentation is not very approachable as it currently is. End-to-end tutorials with a real dataset (or something closely resembling a real dataset) are needed for all applications. How are the output files formatted and how might the user further process those files to address biological questions? For example, it seems that one could use ngsJulia to generate a matrix of genotype calls across samples and loci, starting from a set of mpileup files, but from the documentation I have no idea how to accomplish this task.

 There are eleven different functions for estimating genotype log likelihoods, depending on ploidy, with four different functions for haploids. This strikes me as poor software design. At minimum the eight calcGenoLogLikeX_MajorMinor functions could be collapsed into one, with ploidy as an added argument to the function. Having a single function would make it much easier to update the code and ensure that there aren’t any bugs that prevent it from working consistently across ploidy levels. The hard-coding of tuples for every possible genotype, and the repetitive if-else statements, could be replaced with a more programmatic approach. The programmatic approach with ploidy as a function argument would also enable the software to be used for organisms with ploidy greater than octoploid.

 If I understand correctly, the SNP calling algorithm only seems to work on haploid samples, which would exclude it from being used in most studies. Please clarify.

 The estimation of ploidy from sequencing data using the ngsPloidy is a valuable addition. As an author of other software for marker analysis in polyploids, I frequently hear requests for such functionality, so I expect ngsPloidy to get a lot of use. It would be helpful to include a comparison to other tools such as ploidyNGS, ConPADE, and/or SuperMASSA. Additionally, the algorithm is only tested on a simulated dataset, and I would like to see it tested on an empirical dataset (with flow cytometry used as ground truth), both to see how well it scales up to thousands of markers, and how well it deals with potentially messy data.

 The authors use the term “aneuploidy” incorrectly. Aneuploidy refers to different chromosomes having different copy numbers within one individual. The authors seem to mean different individuals within a population having different ploidies. “Multiploid” is one term for this although depending on the field it is sometimes used as a synonym for “polyploid”.

 How should the user estimate the shape of the site frequency spectrum and the effective population size, which are required inputs for ngsPloidy?

 I tried “Case A” in the ngsPloidy tutorial. It took me a few minutes to figure out the (undocumented) contents of test.A.txt. I assume that columns 6-25 are the true genotypes, column 5 is the allele frequency, and column 26 is the sample allele frequency. The tutorial otherwise worked. However, I didn’t get anything useful when I added the --callGeno flag.

 Similarly to ngsPloidy, ngsPool is only tested on simulated data.

 Minor comments:

- There seems to be a missing right bracket on the left side of equation 2.
- The Figure 2 caption needs to more explicitly define the abbreviations MLE and SAF.
- All of the code to reproduce the analysis in the paper is provided, although there is no documentation explaining the code or indicating which scripts go with which figures.

Are the conclusions about the tool and its performance adequately supported by the findings presented in the article?

Partly

Is the rationale for developing the new software tool clearly explained?

Partly

Is the description of the software tool technically sound?

Partly

Are sufficient details of the code, methods and analysis (if applicable) provided to allow replication of the software development and its use by others?

Partly

Is sufficient information provided to allow interpretation of the expected output datasets and any results generated using the tool?

Partly

Reviewer Expertise:

Bioinformatics, polyploidy, population genetics

I confirm that I have read this submission and believe that I have an appropriate level of expertise to confirm that it is of an acceptable scientific standard, however I have significant reservations, as outlined above.

# Response

**Published:** 2022-11-22

**Authors:** Matteo Fumagalli

**Affiliations:** Queen Mary, University of London, UK

**The manuscript by Mas-Sandoval et al. describes ngsJulia, a collection of functions and scripts in Julia and R for estimating genotype likelihoods from short read DNA sequencing data, as well as estimating allele frequencies in pooled samples, and estimating ploidy where it is unknown. It seems that the goal is also to enable biologists proficient in Julia to develop their own custom genotype calling and population genetic analysis scripts starting with the provided functions for estimating genotype likelihoods. As it is, the manuscript and software suffer from two major flaws: (1) the software and its outputs are not well documented for users or developers, and (2) testing is only performed on simulated data, not empirical data.**

 Thank you for reviewing our manuscript. We addressed all concerns by improving the applicability and documentation of our software and by applying it to empirical data.

**The software documentation is not very approachable as it currently is. End-to-end tutorials with a real dataset (or something closely resembling a real dataset) are needed for all applications. How are the output files formatted and how might the user further process those files to address biological questions? For example, it seems that one could use ngsJulia to generate a matrix of genotype calls across samples and loci, starting from a set of mpileup files, but from the documentation I have no idea how to accomplish this task.**

 We improved the documentation as requested in the specific comments (see below). Examples from simulated mpileup files under various conditions are provided and now clarified. In our response, we clarified that ngsPloidy does not provide a matrix of genotype calls but rather estimates ploidy levels.

**There are eleven different functions for estimating genotype log likelihoods, depending on ploidy, with four different functions for haploids. This strikes me as poor software design. At minimum the eight calcGenoLogLikeX_MajorMinor functions could be collapsed into one, with ploidy as an added argument to the function. Having a single function would make it much easier to update the code and ensure that there aren’t any bugs that prevent it from working consistently across ploidy levels. The hard-coding of tuples for every possible genotype, and the repetitive if-else statements, could be replaced with a more programmatic approach. The programmatic approach with ploidy as a function argument would also enable the software to be used for organisms with ploidy greater than octoploid.**

 We agree with this comment and we now replace all those functions into a unique one that takes ploidy as input parameter (see calcGenoLike function in file functions.jl). We also replaced all if-else statements with a more programmatic approach which makes ngsPloidy (see file ngsPloidy.jl).

**If I understand correctly, the SNP calling algorithm only seems to work on haploid samples, which would exclude it from being used in most studies. Please clarify.**

 The SNP calling algorithm relies on either a likelihood ratio test with the null hypothesis being the pooled allele frequency equal to 0 (similar to Kim et al. 2011 Bioinformatics), or from setting a threshold on the estimated pooled allele frequency. Therefore, it is applicable to all ploidy levels.

**The estimation of ploidy from sequencing data using the ngsPloidy is a valuable addition. As an author of other software for marker analysis in polyploids, I frequently hear requests for such functionality, so I expect ngsPloidy to get a lot of use. It would be helpful to include a comparison to other tools such as ploidyNGS, ConPADE, and/or SuperMASSA. Additionally, the algorithm is only tested on a simulated dataset, and I would like to see it tested on an empirical dataset (with flow cytometry used as ground truth), both to see how well it scales up to thousands of markers, and how well it deals with potentially messy data.**

 This manuscript (submitted as Software Tool Article) aims at proposing a new implementation rather than a new methodology. Additionally, the likelihood function to estimate ploidy levels partly mirrors the approach taken by a parallel study ( https://www.biorxiv.org/content/10.1101/2021.06.29.450340) where we provide extensive comparisons with competing algorithms (including ngsPloidy). Therefore, a comprehensive benchmarking of ngsPloidy against alternative software would be outside the scope of this paper.

 As suggested, we applied ngsPloidy to a real empirical data of isolates from an outbreak of *Candida auris* (Rhodes et al. 2018). Whilst polyploidy could be associated with multidrug-resistant phenotypes, we inferred haploidy for all samples and contigs, in line with findings from the original study. We added a paragraph on both methods and results sections to describe this new analysis.

**The authors use the term “aneuploidy” incorrectly. Aneuploidy refers to different chromosomes having different copy numbers within one individual. The authors seem to mean different individuals within a population having different ploidies. “Multiploid” is one term for this although depending on the field it is sometimes used as a synonym for “polyploid”.**

 We replaced all occurrences of “aneuploidy” in the text and documentation with “multiploidy”.

**How should the user estimate the shape of the site frequency spectrum and the effective population size, which are required inputs for ngsPloidy?**

 The need to specify the shape of the site frequency spectrum and the effective population size is only required when the sample size is too low to have a meaningful estimate of the population allele frequency. Whilst these parameters are not known a prior for nonmodel species, in practice we found that, as long as values are within a reasonable range and the coverage is sufficient, there is not a significant bias associated with inaccurate settings of these parameters. In fact, they simply determine the prior probability of sampling allele frequencies and, for most species and populations, this distribution tends to have more mass at lower frequency values.

**I tried “Case A” in the ngsPloidy tutorial. It took me a few minutes to figure out the (undocumented) contents of test.A.txt. I assume that columns 6-25 are the true genotypes, column 5 is the allele frequency, and column 26 is the sample allele frequency. The tutorial otherwise worked. However, I didn’t get anything useful when I added the --callGeno flag.**

 We now indicate the contents of all columns of the simulated file. The -callGeno flag does not output called genotypes but rather estimate ploidy by first assigning (calling) genotypes, and thus disabling the integrating over unknown genotypes. This is now clarified in the documentation.

**Similarly to ngsPloidy, ngsPool is only tested on simulated data.**

 We now also present an application of ngsPool to empirical data. We reanalysed genomic data from *Arabidopsis* *lyrata* (Fracassetti et al. 2015). In this study, authors generated data both by genotype-by-sequencing and by pooled-sequencing, with the former providing ground-truth values for genotype and allele calls. We found that estimates of minor allele frequencies using ngsPool yield a lower RMSE (root mean squared error) than estimates from Varscan across all SNPs analysed. We added a paragraph both in the methods and results sections to describe these new analyses.

**Minor comments:**

**There seems to be a missing right bracket on the left side of equation 2.**

 Fixed.

**The Figure 2 caption needs to more explicitly define the abbreviations MLE and SAF.**

 We added the acronyms SAF and MLE in the caption.

**All of the code to reproduce the analysis in the paper is provided, although there is no documentation explaining the code or indicating which scripts go with which figures.**

 In https://github.com/mfumagalli/ngsJulia/tree/master/paper we provide all code to replicate the analyses in the paper. As now indicated, ploidy/do.sh would replicate the simulations and estimation of ploidy variation. We now clarify that in the pool and pool/plots folder each folder/script corresponds to the specific figure in the paper.
